# Number of medications and polypharmacy are associated with frailty in older adults: results from the Midlife in the United States study

**DOI:** 10.3389/fpubh.2023.1148671

**Published:** 2023-05-25

**Authors:** Bader Alqahtani

**Affiliations:** Department of Health and Rehabilitation Sciences, Prince Sattam Bin Abdulaziz University, Al-Kharj, Saudi Arabia

**Keywords:** frailty, aging, older, polypharmacy, medications

## Abstract

**Objectives:**

The current study aimed to examine the association between the number of medications, polypharmacy, and frailty in community-dwelling older adults. In addition, the cutoff score for the number of medications related with frailty in this sample was determined.

**Methods:**

A cross-sectional analysis was performed using data of 328 individual aged between 65 and 85 years from the Midlife in the United States (MIDUS 2): Biomarker Project, 2004–2009, a multisite longitudinal study, for 328 individuals aged between 65 and 85 years. All the participants were categorized into two groups based on the number of medications used: no polypharmacy (*n* = 206) and polypharmacy (*n* = 122). The polypharmacy was defined as having 5 or more medication per day. Frailty status was measured using a modified form of Fried frailty phenotype through the presences of the following indicators include low physical activity; exhaustion; weight loss; slow gait speed and muscle weakness. Participants were categorized into three different groups based on total score: 0 as robust, 1 to 2 as prefrail, 3 or more as frail. The relationship between no. of medications, polypharmacy, and frailty was examined using a multinomial logistic regression model. The model was adjusted for age, sex, BMI, and no. of chronic conditions. Receiver operator characteristics and area under the curve were used to determine the cutoff number of medications.

**Results:**

Number of medications, and polypharmacy were associated with being frail (relative risk ratio [RRR]: 1.30; 95% confidence interval [CI]: [1.12, 1.50], *p* = 0.001), (RRR: 4.77; 95% CI [1.69, 13.4], *p* = 0.003), respectively. Number of medications with cutoff 6 medication or more was associated with being in frail category with sensitivity of 62% and specificity of 73%.

**Conclusion:**

Polypharmacy was shown to be significantly related to frailty. A cutoff score of 6 or more medications distinguished frail from non-frail. Addressing polypharmacy in the older population might ameliorate the impact of physical frailty.

## Introduction

Frailty is viewed as a geriatric clinical syndrome that is characterized by a greater vulnerability to outside stressors. Frailty has also been identified as a major health issue for older adults in a growing number of studies. Previous evidence looking at the prevalence of frailty found that 31% of older persons in Oceania, 25% of older adults in Asia, 23% of older adults in the United States, 21% of older adults in Saudi Arabia, and 22% of older adults in Europe had a diagnosis of frailty (1, 2). Frailty has been linked to an increased risk of falling, injuries, prolonged hospital stays, institutionalization, poor quality of life, and higher mortality rate (3). Frailty is defined and measured using a variety of methods, including Fried’s criteria, which defines frailty as having three or more of the following: fatigue, weight loss, weakness, a slow walking speed, and poor physical activity (4). Since decreasing frailty prevalence is a major priority on a global scale, identifying potential risk factors such as polypharmacy, is essential.

Polypharmacy can be defined as using three or four medications or more at the same time (5). Frailty and comorbidities frequently coexist in older adults, are associated with decreasing quality of life and functional ability and result in polypharmacy. Previous studies reported that about 82% of frail older adults have one or more chronic conditions (6). Furthermore, because of their diminished functional reserve and compromised homeostatic compensation mechanisms, older individuals who are more frail may be more likely to encounter negative medication reactions (7). On the other hand, polypharmacy may increase frailty among geriatric population (8).

Previous studies have investigated the relationship between polypharmacy and frailty and determined the optimal number of medications to distinguish between frail and non-frail older adults; however, given the high heterogeneity between these studies in terms of methodologies, sample size, and study cohorts, more research on the relationship between frailty and medications is needed (8). Furthermore, there is a scarcity of research that looked at both the link between polypharmacy and frailty and the cutoff score for the number of medications that was related with frailty. The majority of prior research addressed the relationship within a single geographical region; however, incorporating samples from multiple locations will give further insights into the intricacy of the relationship between frailty and polypharmacy.

The main objective of this study was to evaluate the association between polypharmacy and frailty status in older adults. The secondary objective was to determine the cutoff score for number of medications that was associated with frailty in this population. We hypothesized that frailty is strongly associated with both polypharmacy, and number of medications.

## Methods

### Study design

In this cross-sectional designed study, secondary analyses were performed using data from the Midlife in the United States (MIDUS 2) biomarker project included 1,255 individuals aged between 34 and 84 years from two subsamples: the longitudinal survey sample (*n* = 1,054) and the Milwaukee sample (*n* = 201). The aim of the MIDUS project was to examine the impact of behavioral, psychological, and social aspects in comprehending age-related samples by adding a comprehensive biological assessment to the physical and mental health (9). The participant recruitment and procedure details of this project have been described elsewhere (9). Each subject provided an informed consent, and each MIDUS site received institutional review board approval (9).

### Participants

Individuals (*n* = 328) aged between 65 and 85 years were included in the current study. According to the number prescribed drugs taken, we categorized participants into two groups: no polypharmacy and polypharmacy. Polypharmacy was defined as those using 5 or more medications. No polypharmacy was determined if individuals were used 4 or less medications. Individuals who aged less than 65 years were excluded from the analysis.

### Measures

#### Outcome

Frailty status was measured using a modified form of Fried frailty phenotype through the presences of the following indicators include low physical activity which was assessed by asking the individual if he/she engaged in 20 min of exercise at least three times a week; weight loss; exhaustion [defined by the participants responses as “I felt that everything I did was an effort” and “I could not get going” to questions adopted from the Center for Epidemiological Studies Depression (CES-D) scale]; slow gait speed was assessed using the time (in seconds) required for an individual to walk 50 feet. and muscle weakness, which was measured by grip strength (4). Each criteria was assigned a score of 0 or 1. Participants were categorized into three different groups based on total score: 0 as robust, 1 to 2 as prefrail, 3 or more as frail.

#### Exposure

For the number of medications and polypharmacy, the MIDUS 2 self-administered questionnaire included questions about “Taking prescription medication” and the answer is yes or no, and question about the number of prescribed medications and the answer is numerical. For data analysis, the total number of presently prescribed drugs was used as a continuous variable. According to the numerical data from the second question, the polypharmacy was assessed and defined as those used 5 or more medications. And included in the analysis as a categorical variable. The similar assessment was performed in the previous studies (8).

#### Covariates

All demographical and clinical data were obtained including age, sex, weight, height, and marital status. Age was measured in years. Sex was categorized into males and females. Weight was measured in kilograms and height was measured in centimeters. Then body mass index (BMI) was calculated by dividing the weight in kilograms by the height in m^2^. Number of chronic conditions was obtained during the interviews by asking about the number of chronic conditions (9).

### Statistical analyses

Data were analyzed using statistical software Stata version 15.1 (Stata Corp, College Station, TX). Descriptive data were reported using mean and standard deviation for continuous variables and numbers with percentages for categorical variables. We used *Wilcoxon test* to *compare* group with polypharmacy an without polypharmacy group for continuous variables, and we used Chi-square test to compare categorical variables across polypharmacy groups. A multinomial logistic regression model was constructed to examine the association between polypharmacy using 5 medications or more and less than 5 medications as a reference category. and frailty status, with relative risk ratio (RRR) and 95% confidence intervals (95% CI) were reported. Frailty status has 3 categories: non-frail, prefrail, and frail. Two models were created. Model 1 provides crude estimates. Model 2 was adjusted for age, sex, BMI, and number of chronic conditions. The non-frail group was used as the reference group. Same procedure was used to study the association between number of medications and frailty status.

A binary logistic regression to identify the cut-off, Receiver operating characteristic (ROC) curve with an area under the curve (AUC) were used to identify the cutoff score for number of medications, and to show the overall accuracy of the model in distinguishing frail subjects from the combination into a single category of pre-frail and non-frail subjects. The optimal cutoff score for number of medications was identified using largest Youden index (sensitivity + [1 – specificity]). An alpha level of 0.05 was used for all models in the analysis.

## Results

**Figure 1:**
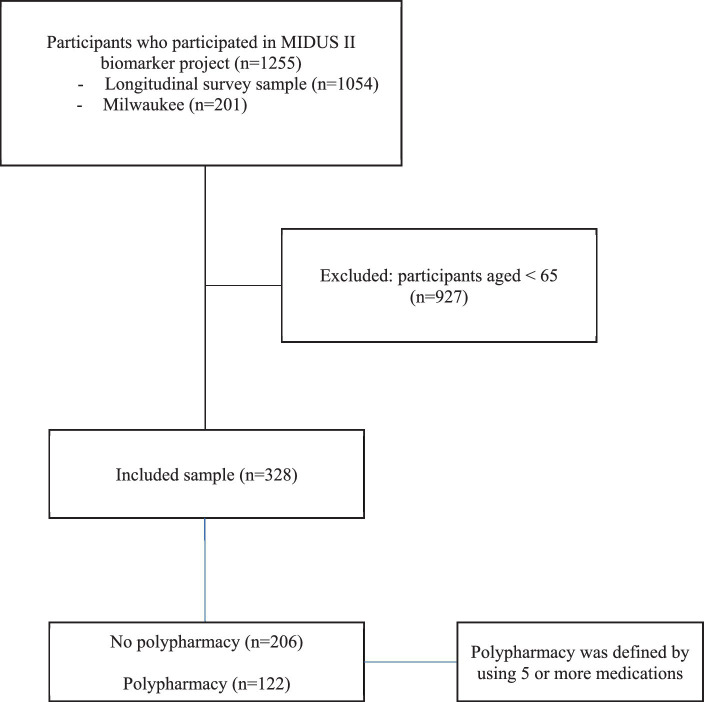
Flow chart of participants’ enrollment.

**Figure 2:**
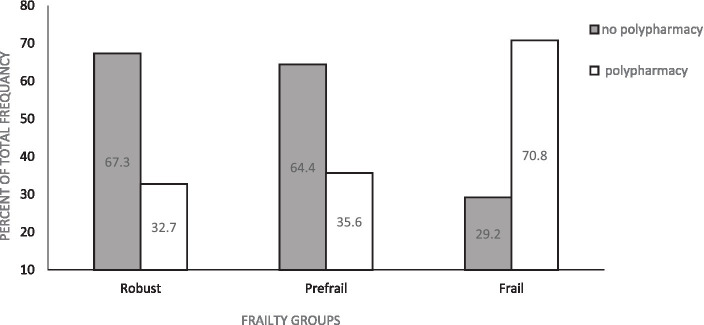
Distribution pattern of polypharmacy across frailty groups.

A total of 328 participants were included in the current study see Figure 1. The prevalence of polypharmacy and demographics and clinical variables for included participants are shown in Table 1. Of these variables, number of chronic diseases, BMI, gait speed, frailty, and number of medications were significantly different between participants with polypharmacy compared to those without polypharmacy. In addition, about 70% of frail participants and 35% of prefrail participants had polypharmacy (Figure 2).

**Table 1:** Participant demographics and clinical characteristics (*n* = 328).

Variable	Total	No polypharmacy (≤4)	Polypharmacy (≥5)	*p*-value
Age (y)	72.6 (5.8)	72.6 ± 5.8	72.7 ± 6	0.828
Gender, *n* (%)				0.542
Men	148	93 (62.8)	55 (37.2)	
Women	180	113(62.7)	67 (37.3)	
Marital status, *n* (%)				0.580
Single	14	10 (71.4)	4 (28.6)	
Married	210	133 (63.3)	77 (36.6)	
Divorced	46	29 (63.1)	17 (36.9)	
Widowed	58	33 (56.9)	25 (43.1)	
Number of comorbidities, *n* (%)				<0.001
None	6	6 (100)	0	
1	15	15 (100)	0	
2 or more	307	185 (60.3)	122 (39.7)	
Number of medications, mean (SD)	4 (3.1)	2 (1.3)	7.3 (2.3)	<0.001
BMI^*^ (kg/m^2^), mean (SD)	28.9 (5.2)	28.1(5.01)	30.4 (5.3)	<0.001

BMI: body mass index; SD, standard deviation. According to Fried frailty phenotype.

Table 2 shows the association between number of medications and polypharmacy with frailty categories using multinominal logistic regression. Crude model shows that increased in number of medications, and polypharmacy were associated with being frail (RRR: 1.31; 95% CI [1.14, 1.50], *p* = 0.001), (RRR: 4.99; 95% CI [1.89, 13.1], *p* < 0.001), respectively. Model 2 shows that increased in number of medications were associated with frail category (RRR: 1.30; 95% CI [1.12, 1.50], *p* = 0.001) after adjustments for age, sex, BMI, and number of chronic conditions. Polypharmacy was associated with frail category (RRR: 4.77; 95% CI [1.69, 13.4], *p* = 0.003) after adjustments for age, sex, BMI, and number of chronic conditions.

**Table 2:** Multinomial logistic regression showing the association between number of medications and polypharmacy and frailty categories.

Fried frailty index	Model 1^1^ (*N* = 328)			Model 2^2^ (*N* = 328)		
	RRR (95% CI)			RRR (95% CI)			Non-frail	Pre-frail	Frail	Non-frail	Pre-frail	Frail
Number of medications*	Reference	1.08 (0.99,1.17)	1.31 (1.14, 1.50)	Reference	1.02 (0.93,1.12)	1.27 (1.09, 1.47)
Polypharmacy	Reference	1.13 (0.69,1.86)	4.99 (1.89, 13.1)	Reference	0.88 (0.50, 1.52)	4.02 (1.40, 11.5)

RRR, relative risk ratio. *Included in the analysis as a continuous variable. ^1^Model 1 unadjusted model. ^2^Model 2 adjusted for age, sex, BMI and number of chronic conditions.

**Figure 3:**
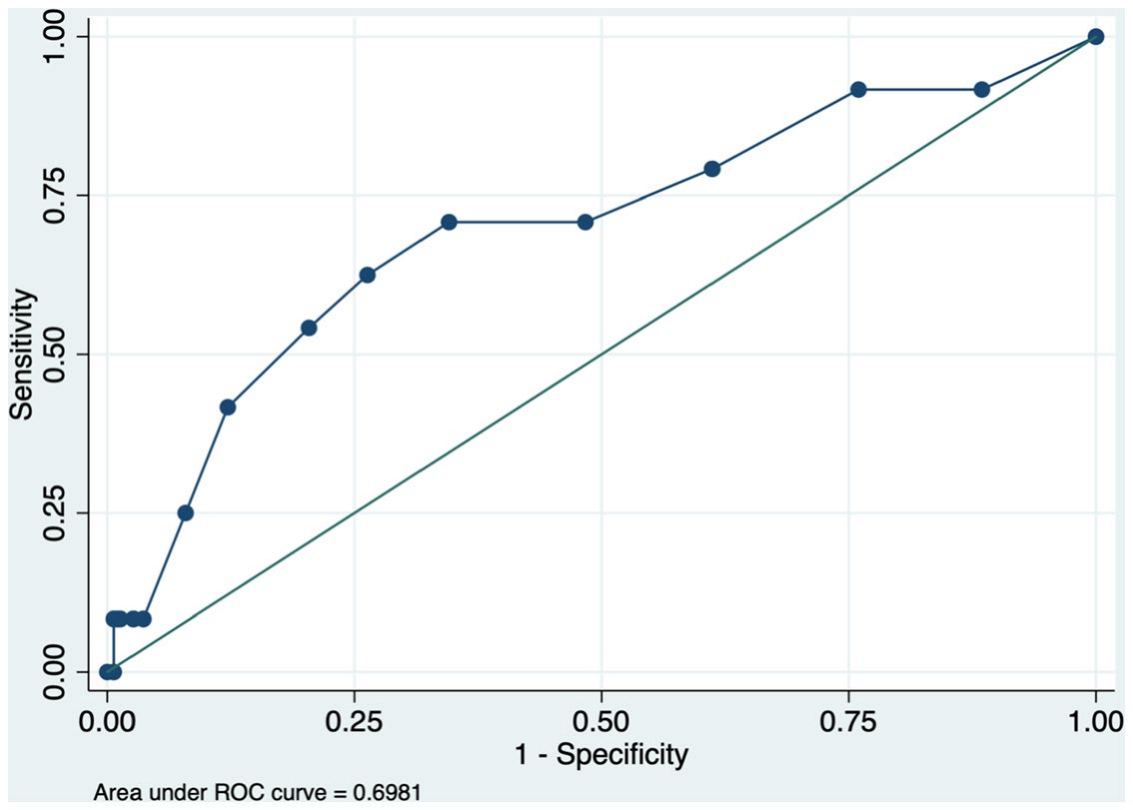
Receiver operating characteristic (ROC) for number of medications associated with frailty.

Table 3 shows the ROC and AUC for number of medications associated with frailty category. Number of medications with cutoff 6 medication or more was associated with being in frail category with sensitivity of 62% and specificity of 73% (Figure 3).

**Table 3:** Receiver operating characteristic (ROC) curve and cut-off score for number of medications associated with frailty.

Variables	AUC (95% CI)	Cut-off scores (sensitivity, specificity) *
Number of medications	0.69 (0.57, 0.82)	≥6 (0.62, 0.73)
		≥5 (0.69, 0.65)
		≥4 (0.70, 0.51)

^*^Ordered by highest Youden index.

## Discussion

The study examined the association between number of medications and polypharmacy with frailty among older adults. A bout 37% of the current sample had polypharmacy The current study found that number of medications and polypharmacy were associated with frailty in this population, also after adjustments for age, sex, BMI, and number of chronic medications. This study identified the cutoff number of medications that distinguished frail from non-frail, which was 6 medications or more with good sensitivity and specificity.

The results of the current study found that an increase in number of medications was associated with a higher risk for frailty. These findings were consistent with previous cross-sectional and longitudinal studies that found strong association between number of medications and frailty among older adults (5, 10–12). In addition, our findings were similar to previous studies that found a significant association between polypharmacy and frailty (11, 13, 14). Our research supports the association between polypharmacy and frailty even after controlling for potential confounders.

Polypharmacy may influence the onset of frailty through a number of plausible mechanisms. Many clinical components or aspects of frailty, such as weight loss, balance difficulties, poor nutritional status, or functional decline, have been directly related with the polypharmacy. It has been suggested that a numerous clinical indicator of frailty, such as weight loss, balance issues, poor nutrition, or disability, have been strongly correlated with the number of medicines taken (5). As frailty and the number of chronic diseases is associated, and polypharmacy is common with many chronic conditions, this could explain the association between polypharmacy and frailty. However, in the current study, the association between polypharmacy and frailty persisted after adjusting for the number of chronic conditions, which may indicate that polypharmacy may contribute to the frailty through medication side effects (11).

In the present study, a cutoff number for used medications to predict frail status was estimated. The ROC analysis found that 6 or more medications is a cutoff that can discriminate frail from non-frail with good sensitivity and specificity. Our finding was similar to previous work that they found that using 6 or more medications was associated with frailty (11, 14, 15). Gnjidic et al. established a 6.5 as a cutoff number of medications that can predict the incidence of frailty (16). In the aforementioned study, a sample was composed of 70 years and older community-dwelling males, while in our study we included both male and female and a younger age group. Furthermore, taking 6 medications or more have been also found to be associated with increased adverse effect of medications, as well as increased risk of falls (17, 18). Which make it more rational to consider the cutoff of 6 or more medications as a predictor of frailty. Moreover, although 6 medications as a cut-off yielded a better Youden index score, in terms of sensitivity and specificity there was small difference between the cut-off 5 and 6 medications and that can be justified by including only prescribed medications in the analysis.

The present research provides insight into the association between frailty and the number of medications and polypharmacy, as well as a cut-off number of medications that can distinguish frail from non-frail older adults. However, there are several limitations should be considered and addressed in future research. First, this study is a cross-sectional study which limits the ability to establish a causal relationship between polypharmacy and frailty among older adults, since a longitudinal study design to establish causality is needed. Second, different types of medications, such as cancer medications, were not included in the self-reported questions. Third, types of medications, medication dosage, adherence to medications, participants’ conditions (healthy or diseased), were not included in the analysis, so sensitivity analysis on these factors should be considered in future studies. Finally, the current sample was recruited from United States (US), therefore the generalizability of our findings may be limited to older adults living in the US.

## Conclusion

Among older adults, the number of medications and polypharmacy with 6 or more medications might be significantly linked to frail compared to non-frail older adults. Addressing polypharmacy in the older population might ameliorate the impact of frailty. Future work should examine the types of medications that might be associated with frailty status.

## Data availability statement

The original contributions presented in the study are included in the article/supplementary files, further inquiries can be directed to the corresponding author.

## Ethics statement

Ethical review and approval was not required for the study on human participants in accordance with the local legislation and institutional requirements. Written informed consent for participation was not required for this study in accordance with the national legislation and the institutional requirements.

## Author contributions

BA conceived and designed the study, data analysis and interpretation, and review and wrote the final version of the manuscript.

## Funding

This study was supported via funding from Prince Sattam bin Abdulaziz University project number (PSAU/2023/R/1444).

## Conflict of interest

The author declares that the research was conducted in the absence of any commercial or financial relationships that could be construed as a potential conflict of interest.

## Publisher’s note

All claims expressed in this article are solely those of the authors and do not necessarily represent those of their affiliated organizations, or those of the publisher, the editors and the reviewers. Any product that may be evaluated in this article, or claim that may be made by its manufacturer, is not guaranteed or endorsed by the publisher.
